# Implementation of a roadmap for the comprehensive diagnosis, follow-up, and research of childhood leukemias in vulnerable regions of Mexico: results from the PRONAII Strategy

**DOI:** 10.3389/fonc.2024.1304690

**Published:** 2024-04-03

**Authors:** Juan Carlos Núñez-Enríquez, Rubí Romo-Rodríguez, Pedro Gaspar-Mendoza, Gabriela Zamora-Herrera, Lizeth Torres-Pineda, Jiovanni Amador-Cardoso, Jebea A. López-Blanco, Laura Alfaro-Hernández, Lucero López-García, Arely Rosas-Cruz, Dulce Rosario Alberto-Aguilar, César Omar Trejo-Pichardo, Dalia Ramírez-Ramírez, Astin Cruz-Maza, Janet Flores-Lujano, Nuria Luna-Silva, Angélica Martínez-Martell, Karina Martínez-Jose, Anabel Ramírez-Ramírez, Juan Carlos Solis-Poblano, Patricia Zagoya-Martínez, Vanessa Terán-Cerqueda, Andrea Huerta-Moreno, Álvaro Montiel-Jarquín, Miguel Garrido-Hernández, Raquel Hernández-Ramos, Daniela Olvera-Caraza, Cynthia Shanat Cruz-Medina, Enoch Alvarez-Rodríguez, Lénica Anahí Chávez-Aguilar, Wilfrido Herrera-Olivares, Brianda García-Hidalgo, Lena Sarahí Cano-Cuapio, Claudia Guevara-Espejel, Gerardo Juárez-Avendaño, Juan Carlos Balandrán, Ma. del Rocío Baños-Lara, Mariana Cárdenas-González, Elena R. Álvarez-Buylla, Sonia Mayra Pérez-Tapia, Diana Casique-Aguirre, Rosana Pelayo

**Affiliations:** ^1^ Unidad de Investigación Médica en Epidemiología Clínica, Unidad Médica de Alta Especialidad (UMAE) Hospital de Pediatría “Dr. Silvestre Frenk Freund” Centro Médico Nacional Siglo XXI, Instituto Mexicano del Seguro Social, Mexico City, Mexico; ^2^ Laboratorio de Citómica del Cáncer Infantil, Centro de Investigación Biomédica de Oriente, Delegación Puebla, Instituto Mexicano del Seguro Social, Puebla, Mexico; ^3^ Consejo Nacional de Humanidades, Ciencias y Tecnologías (CONAHCYT), Mexico City, Mexico; ^4^ Facultad de Medicina, Benemérita Universidad Autónoma de Puebla (BUAP), Puebla, Mexico; ^5^ Instituto de Fisiología, Benemérita Universidad Autónoma de Puebla (BUAP), Puebla, Mexico; ^6^ Facultad de Ciencias Biológicas, Benemérita Universidad Autónoma de Puebla (BUAP), Puebla, Mexico; ^7^ Facultad de Ciencias Químicas. Benemérita Universidad Autónoma de Puebla, Puebla, Mexico; ^8^ Unidad de Desarrollo e Investigación en Bioterapéuticos (UDIBI), Escuela Nacional de Ciencias Biológicas, Instituto Politécnico Nacional, Mexico City, Mexico; ^9^ Laboratorio Juárez, Medicina de Laboratorio Clínico de Alta Especialidad, Biología Molecular e Investigación Clínica, Oaxaca de Juárez, Oaxaca, Mexico; ^10^ Servicio de Hemato-Oncología Pediátrica, Hospital de la Niñez Oaxaqueña, Secretaría de Salud, Oaxaca, Mexico; ^11^ Servicio de ONCOCREAN, Hospital General de Zona 01, Delegación Oaxaca, Instituto Mexicano del Seguro Social, Oaxaca, Mexico; ^12^ Servicio de Hematología, Unidad Médica de Alta Especialidad, Hospital de Especialidades “Manuel Avila Camacho”, Instituto Mexicano del Seguro Social, Puebla, Mexico; ^13^ Departamento de Oncología. Hospital para el Niño Poblano. Secretaría de Salud, Puebla, Mexico; ^14^ Servicio de Oncohematología Pediátrica, Instituto de Seguridad y Servicios Sociales de los Trabajadores al Servicio de los Poderes del Estado de Puebla (ISSSTEP), Puebla, Mexico; ^15^ Servicio de Hematología Pediátrica, Instituto de Seguridad y Servicios Sociales de los Trabajadores del Estado (ISSSTE), Puebla, Mexico; ^16^ Servicio de Oncohematología Pediátrica, Hospital General del Sur, Puebla, Mexico; ^17^ Servicio de Oncología Pediátrica, Hospital Infantil de Tlaxcala, Tlaxcala, Mexico; ^18^ Department of Pathology, New York University (NYU) School of Medicine, New York, NY, United States; ^19^ Centro de Investigación Oncológica, Una Nueva Esperanza, Universidad Popular Autónoma del Estado de Puebla, Puebla, Mexico; ^20^ Laboratorio Nacional para Servicios Especializados de Investigación, Desarrollo e Innovación (I+D+i) para Farmoquímicos y Biotecnológicos, LANSEIDI-FarBiotec-CONACyT, Mexico City, Mexico; ^21^ Departamento de Inmunología, Escuela Nacional de Ciencias Biológicas, Instituto Politécnico Nacional, Mexico City, Mexico; ^22^ Unidad de Educación e Investigación, Instituto Mexicano del Seguro Social, Mexico City, Mexico

**Keywords:** childhood leukemias, Mexico, measurable residual disease (MRD), diagnosis, vulnerable regions

## Abstract

The main objective of the National Project for Research and Incidence of Childhood Leukemias is to reduce early mortality rates for these neoplasms in the vulnerable regions of Mexico. This project was conducted in the states of Oaxaca, Puebla, and Tlaxcala. A key strategy of the project is the implementation of an effective roadmap to ensure that leukemia patients are the target of maximum benefit of interdisciplinary collaboration between researchers, clinicians, surveyors, and laboratories. This strategy guarantees the comprehensive management of diagnosis and follow-up samples of pediatric patients with leukemia, centralizing, managing, and analyzing the information collected. Additionally, it allows for a precise diagnosis and monitoring of the disease through immunophenotype and measurable residual disease (MRD) studies, enhancing research and supporting informed clinical decisions for the first time in these regions through a population-based study. This initiative has significantly improved the diagnostic capacity of leukemia in girls, boys, and adolescents in the regions of Oaxaca, Puebla, and Tlaxcala, providing comprehensive, high-quality care with full coverage in the region. Likewise, it has strengthened collaboration between health institutions, researchers, and professionals in the sector, which contributes to reducing the impact of the disease on the community.

## 1 Introduction

Acute leukemia (AL) has emerged as a major global health concern because it has the highest incidence rate among pediatric neoplasms, accounting for 28.2% of the overall incidence of cancer worldwide among children and adolescents aged < 19 years (1). Among the various subtypes of childhood leukemia, B-cell precursor acute lymphoblastic leukemia (B-ALL) is the most common, followed by acute myeloid leukemia (AML). T-cell precursor acute lymphoblastic leukemia (T-ALL) represents approximately 10% of all pediatric cases (2–6). Despite progress made through innovative therapies and treatment regimens, achieving disease-free survival rates of 90% in developed countries, hematological malignancies in children persist as the leading cause of disease-related deaths globally (1, 7).

In Mexico, the frequency of childhood leukemias, particularly, the incidence of ALL is among the highest reported in the world (8). Moreover, its mortality rate is 1.8 times higher than the global average (1). Within this oncological landscape, B-ALL is the most common, accounting for approximately 85% of ALL cases (9). The number of AML cases in children and adolescents in Oaxaca has increased and has been reported in 22.48% of AML cases and 70.64% of B-ALL cases. However, what stands out in AML cases, 65.31% of patients presented with genetic aberrations, a much higher frequency than the 18.83% of genetic anomalies found in patients with B-ALL. This high percentage of genetic alterations in AML is of particular concern and significantly different from that in B-ALL (10). A recent study conducted in Mexico City reported that the incidence of AL is 63.3 (cases per million), 53.1 for ALL, and 9.4 for AML, placing them among the highest recorded globally (8). Mexico’s incidence rate of AL surpasses that of many other countries, and Hispanics living in the United States have a particularly challenging prognosis (11–13). This disparity could be attributed to several factors, including modifications in diagnostic methodologies, changes in disease classification, environmental and lifestyle factors, genetic components, and ethnic and racial variance (14). It is imperative that all these aspects be considered in strategies aimed at identifying the etiology of AL and improving the prognosis of Mexican and other pediatric populations of Hispanic origin diagnosed with this disease.

Since 2021, the National Project for Research and Incidence of Childhood Leukemias, known by its spanish acronym PRONAII, has aimed to reduce early deaths in children with leukemia from vulnerable regions of the country, specifically, in the states of Oaxaca, Puebla, and Tlaxcala. It focuses on contextualized scientific research to propose innovative approaches for the control of AL, impacting clinical practice and public policies.

In Mexico City, where the main public pediatric hospitals in the country are located, with the best infrastructure available for the diagnosis and treatment of patients with AL, a high frequency of early relapses and deaths has been reported and efforts have been made to achieve better survival rates (15). Nonetheless, there were other regions in the country where this scenario is not observed, resulting in delayed diagnosis and a lack of timely treatment opportunities. According to official data from the National Council for Evaluation of Social Development Policy (CONEVAL) in 2018, Oaxaca, Puebla and Tlaxcala were considered among the most vulnerable regions of our country from a socioeconomic perspective. Specifically, they presented a high percentage of the population living in poverty: 66.4% in Oaxaca, 58.9% in Puebla, and 48.4% in Tlaxcala, reflecting the urgency of targeted interventions. In parallel, limited access to health services (16.3% in Oaxaca, 20.8% in Puebla, and 13.7% in Tlaxcala) (16) highlights the need to strengthen the infrastructure and health services.

In most of the states of our country, many public hospital centers do not have essential phenotyping services to adequately diagnose and classify AL. It is important to emphasize that it is estimated that 99.3% of the Mexican patients with childhood leukemia are attended in public hospitals. Likewise, they lack effective disease monitoring using MRD. Diagnoses in these states usually depend on institutional resources, and typing studies are sometimes referred to as private laboratories. This, in the absence of following a standardized protocol could lead to a uniformity in the diagnoses and impact in the classification and treatment assignment of each patient with leukemia. Furthermore, there is no standardized system for handling samples from hospitals in the diagnostic laboratories.

Accurate classification, appropriate treatment assignment, and constant monitoring have been shown to enhance the effectiveness of treatment in patients with childhood leukemia from different parts of the world, particularly in developed countries, where resources are sufficient. It is well known that the detection of MRD is the most significant predictor of unfavorable outcomes in patients with AL (17–24); however, there are other factors, such as molecular abnormalities, which are also important prognostic factors in AL. Moreover, an early diagnosis and timely initiation of treatment in children with acute leukemia translate to higher survival rates. Long periods between symptom onset and diagnosis confirmation, immunophenotype classification, and treatment initiation may compromise the overall survival of children with AL (25).

Therefore, addressing the high rates of relapse and early mortality associated with AL is a daunting challenge, underscoring the indispensable role of the PRONAII Childhood Leukemia in both medicine and the Mexican society. This project uses advanced scientific, technological, and social tools to provide personalized preventive and curative solutions to reduce the risk of relapse and early deaths in children with AL from vulnerable regions of Mexico (26). One of the pillar strategies of PRONAII Childhood Leukemia is the optimal functioning of the roadmap. This includes both the central urban areas and rural communities from Oaxaca, Puebla, and Tlaxcala. Through a systematized algorithm, starting from the patient’s arrival at the hospital to the issuance of the standardized report to the treating physician. The purpose was to guarantee a timely, complete, and continuous diagnosis and follow-up for each girl, boy, and adolescent with suspected childhood leukemia.

## 2 Methods

### 2.1 Essential elements of the intervention

**Figure 1:**
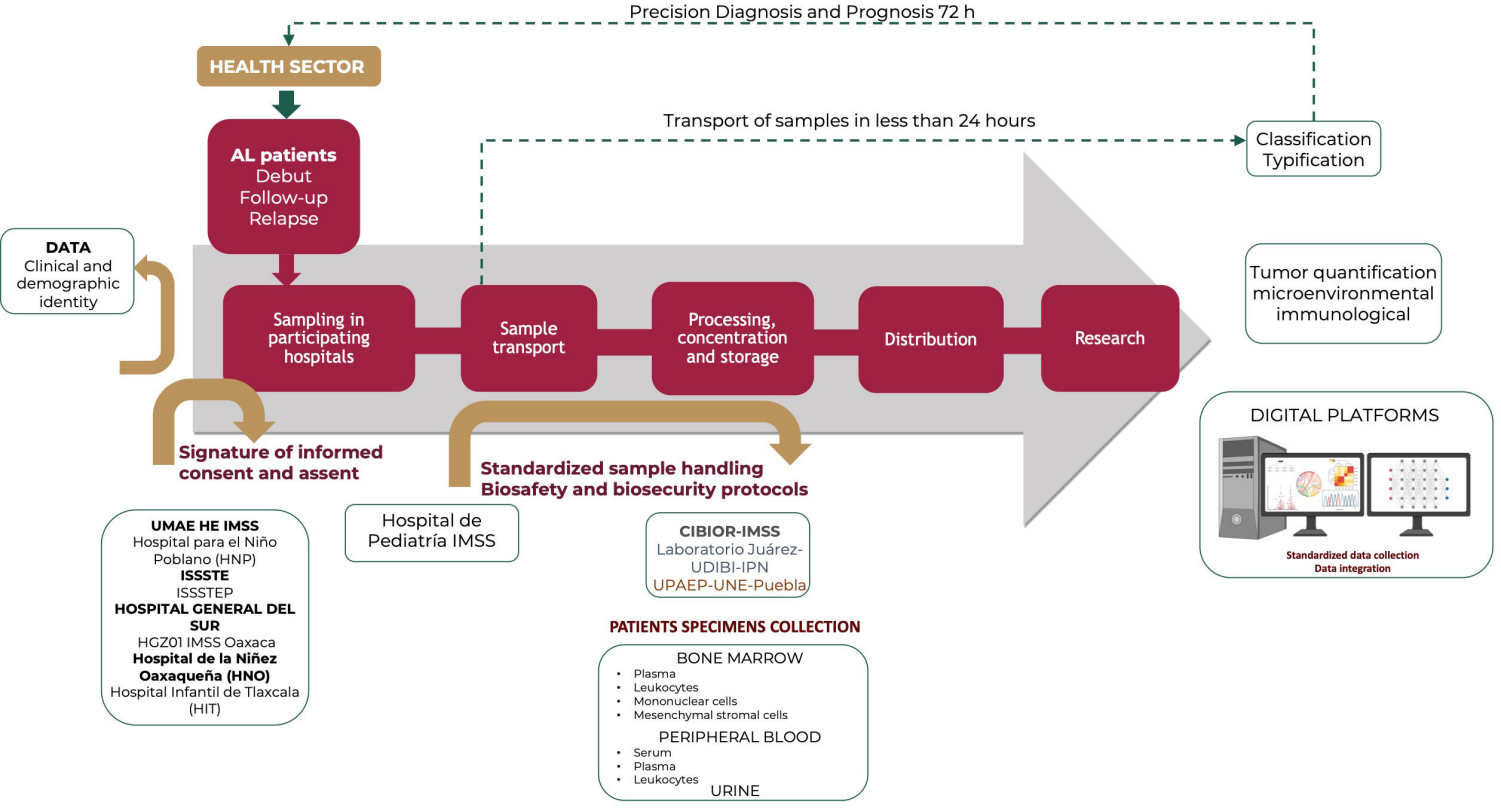
A roadmap for leukemia in vulnerable regions: Custody from clinical suspicion to comprehensive treatment. The process was structured into seven crucial phases that guaranteed the meticulous treatment of each leukemia patient sample. Furthermore, these stages ensure the adequate concentration, management, and analysis of the information produced. This approach supports accurate patient follow-up, focused research, and data-driven clinical decision making. In Puebla, the hospitals that collaborated in this process are the Unidad Médica de Alta Especialidad Hospital de Especialidades “Dr. Manuel Ávila Camacho” Hospital of the Instituto Mexicano del Seguro social (IMSS), the Hospital para el Niño Poblano (HNP) of the Ministry of Health, the Instituto de Seguridad y Servicios Sociales para los Trabajadores del Estado (ISSSTE), the Instituto de Seguridad y Servicios Sociales para los Trabajadores del Estado de Puebla (ISSSTEP) and the Hospital General del Sur. In Tlaxcala, collaboration came from the Hospital Infantil de Tlaxcala (HIT). In Oaxaca, the Hospital de la Niñez Oaxaqueña (HNO) of Instituto de Salud para el Bienestar (INSABI), and the Hospital General de Zona No. 1 (HGZ01) of the IMSS Oaxaca actively participated.

Through an inter-institutional effort, the PRONAII Childhood Leukemia Incidence Roadmap was established, involving a collaborative network of approximately 50 specialists from various disciplines linked to the field of health. As shown in **Figure 1**, the pathway comprises multiple stages that are carefully systematized.

### 2.2 Health sector and patients

The process begins in the Health Sector, where hematologists and oncologists specialized in childhood cancer identify and initiate studies to confirm or rule out the diagnosis of leukemia in patients who present with signs, symptoms, and alterations in laboratory tests related to the disease, as well as in those who have already been diagnosed but require a follow-up analysis to monitor the response to treatment. Additionally, patients who experienced relapse of leukemia were included in this study.

### 2.3 Collection of clinical and demographic data

To ascertain on each participating hospital the PRONAII Childhood Leukemia Epidemiology Group is responsible for systematically recording all new cases of leukemia following international recommendations to conduct a population-based cancer registry. Patient parents or guardians were required to sign an informed consent form to authorize the use of data and samples for research. This process respects confidentiality by the assignment of a unique folio, which is a sequential numerical code that does not initially reveal his/her identity. To collect demographic and clinical information, face-to-face interviews with parents/guardians of children confirmed with AL are conducted by trained personnel. Data were entered into an electronic form that is part of the Clinical and Demographic Identity Database. This form includes variables such as patient sex, age at the time of diagnosis, municipality of residence, and socioeconomic level to facilitate epidemiological analysis (27). The digital platform used for this process was configured to allow access only to authorized researchers, thus ensuring the confidentiality and security of the information.

### 2.4 Folio number assignment

The folio consists in a sequential numerical code that was not directly linked to the individual’s identity in the initial phase. It comprises three numerical elements, the first of which indicates whether the patient has Down Syndrome. The number “1” is used to indicate patients without Down Syndrome and the number “3” indicates those who have it. The second element is the geographic location code, which reflects the state of the Mexican Republic in which patients receive medical care. The codes are specific for each state, assigning “1” for the State of Oaxaca, “2” for the State of Puebla and “3” for the State of Tlaxcala. Finally, folio includes a unique consecutive number for each patient within their respective statuses. Thus, folio 31145 specifically identifies a patient with Down Syndrome who is being treated in the state of Oaxaca and is the 145th patient registered in that state. This folio is essential for tracking the sample and related data such as clinical, demographic, and laboratory results.

### 2.5 Sampling

Bone marrow (BM) aspiration is performed by pediatric hematologists/oncologists who rigorously adhere to the quality and safety standards of medical care (**Figure 1**). It should be noted that each of these institutions validated and approved the research protocol through their Ethics and Research Committees. BM, urine, and peripheral blood samples were obtained at the time of diagnosis. Additional BM collection was performed at two critical treatment times: during the remission phase and in cases of disease relapse. Between March 2022 and August 2023, a total of 528 BM samples were collected from 345 pediatric patients to diagnose AL. The sex distribution was 193 boys and 152 girls, with ages ranging from 1 month to 19 years old. The median age of the patients was 10 years.

### 2.6 Sample transport

With the collaboration of properly trained personnel, BM samples are being sent to the Childhood Cancer Cytomics Laboratory, located at the Centro de Investigación Biomédica de Oriente (CIBIOR) of the IMSS. Sample shipping logistics is under the supervision of a specialized field coordinator, who ensures that each sample is transported within a triple packaging system to ensure its integrity and biological containment. This packaging complies with the national and international biosafety regulations for the handling and transportation of infectious substances. Moreover, a transporter is required to record the shipment details of a sample in a digital form. This documentation is crucial because it enables receiving laboratories to prepare for the sample’s proactive arrival. Such preparedness is key to ensuring seamless transition, preserving the chain of custody, and upholding the integrity of the sample at every stage.

To guarantee adequate tracking of the samples, two electronic forms were implemented that allowed real-time monitoring from the moment they left the hospital until they were received in the laboratory:

Sample output electronic form: This electronic form records crucial information, such as the patient’s Folio, the hospital of origin, the name of the treating physician, the date of collection, demographic details of the patient (age and sex), state of origin, type of analysis, immunophenotype or MRD requested by the hematologist, details about the type and quantity of sample, and, finally, the person in charge of transportation logistics.

The sample arrival electronic form contains details such as the Folio, laboratory that receives the sample, type of sample, and names of the people in charge of both transporting and receiving the sample.

These tracking systems ensure that each sample can be tracked and handled appropriately, reducing the risk of loss or confusion, and strengthening the confidence in the results obtained.

Delivery was guaranteed within a period of no more than 24 h to ensure the integrity and quality of the material, which is essential for the reliability of the results. Samples were not collected and shipped on holidays to ensure that their viability was not compromised. Once in the laboratory, sample typing was performed according to the instructions of a specialist hematologist. Depending on the patient’s clinical evaluation, a specialist may request immunophenotype analysis for diagnostic purposes or an MRD test to monitor disease progression.

### 2.7 Classification of acute leukemia by immunophenotyping

To identify and characterize acute leukemia, BM samples were stained, and EuroFlow™ antibody panels were used to detect normal, reactive, and malignant leukocytes (28) (**Table 1**). Additionally, to evaluate the treatment response, high-sensitivity MRD tests were performed by analyzing at least five million cells to ensure accuracy. This process utilizes the same antibody panels as the initial diagnosis, except for B-ALL, in which a specific panel is employed (29). The flow cytometers were standardized following the EuroFlow™ Standard Operating Protocol (SOP) for the configuration and compensation of the instruments. BD FASCCanto™ II cytometers (San Jose, CA, USA) equipped with BD FACSDivA™ software and BD FACSLyric™ cytometer equipped with BD FACSuite™ software were used. (www.euroflow.org; accessed September 12, 2023) (30). The Infinicyt™ software (version 2.0, Cytognos SL, Salamanca, Spain) was used for multiparametric cytometric analysis. The strategy for the detection and classification of acute leukemia through immunophenotyping using flow cytometry comprises of two fundamental stages. First, the suspicious sample was stained using an 8-color antibody panel (Acute Leukemia Orientation Tube, ALOT) that allowed the identification and enumeration of all hematopoietic cell populations, while enabling the determination of the lineage of the affected precursor cells. Subsequently, once the lineage of leukemic cells had been defined, multitube panels of antibodies were used to allow complete characterization and classification. These cells were classified into five groups according to their immunophenotype: ProB ALL (CD34^+^ CD19^+^ cyCD79a^+^), ProB-PreB ALL (CD34^-/+^ CD19^+^ cyCD79a^+^), PreB ALL (CD34^-^ CD19^+^ cyCD79a^+^), T ALL (cyCD3^+^ smCD3^lo^ CD7^+^), and AML (cyMPO^+^ or CD7^+^). By comparing the immunophenotypic profiles of the blast cells with those of their normal counterparts, it was possible to identify their maturation stage and aberrant phenotypes, such as lineage infidelity, asynchronous antigen expression, and absence or overexpression of markers. Leukemia was diagnosed by pediatric hematologist-oncologists based on a comprehensive evaluation. This included an assessment of clinical presentations, detailed analysis of BM aspirate, examination of cell morphology, immunophenotyping, and genetic characteristics, following the criteria established by the 2008 World Health Organization (WHO) classification for these disorders.

**Table 1:** EuroFlow™ antibody panels for immunophenotyping acute leukemia.

Marker	B-ALL	T-ALL	AML
CD45	X	X	X
CD34	X	X	X
CD19	X		X
cyCD79a	X		
cyCD3		X	
CD7		X	X
smCD3		X	
cyMPO			X
CD20	X		
CD58	X		
CD66c	X		
CD10	X	X	X
CD38	X		X
smIgκ	X		
cyIgμ	X		
CD33	X		X
CD117	X	X	X
smIgM	X		
smIgλ	X		
CD9	X		X
nuTdT	X	X	X
CD13	X		X
CD22	X		X
CD24	X		
CD21	X		
CD15	X		X
NG2	X		X
CD123	X		X
CD81	X		
CD2		X	
CD1a		X	
CD5		X	
CD8		X	
CD99		X	
CD4		X	X
HLA-DR			X
CD16			X
CD11b			X
CD35			X
CD64			X
CD300e			X
CD14			X
CD36			X
CD105			X
CD71			X
CD56			X
CD42a/CD61			X
CD203c			X
CD41			X
CD25			X
CD42b			X

Blast cells were discerned through the identification of the immaturity markers CD45 and CD34. AL cases were systematically classified into five categories based on the specific cell lineage affected: ProB-ALL (CD34_+_CD19_+_cyCD79a_+_), ProB-PreB-ALL (CD34_-/+_CD19_+_cyCD79a_+_), PreB-ALL (CD34_-_CD19_+_cyCD79a_+_), T-ALL (cyCD3_+_smCD3loCD7_+_), and AML (cyMPO_+_ or CD7_+_cyCD3_-_). Complementary markers were used for comprehensive characterization to further delineate the blast population. AL, Acute leukemia; ALL, Acute lymphoblastic leukemia; AML, Acute myeloid leukemia; CD, cyCD79a; cy, cytoplasmic; sm, surface membrane; nu, nuclear. The highlighted markers correspond to those associated with the lineage of the blast cells, while the colored markers indicate the maturation stage.

### 2.8 PRONAII childhood leukemia cell repository

This roadmap enabled the creation of a cell repository that groups several components extracted from three different samples from the same patient. The remaining BM samples used for diagnosis as well as the patient’s peripheral blood (PB) and urine samples were meticulously processed to obtain various blood and cell components.

#### 2.8.1 Sample processing

BM was collected in BD Vacutainer^®^ tubes containing EDTA (purple caps). Whole bone marrow (WBM) was stored, blood plasma (BP) was obtained by centrifugation, and leukocytes were isolated using a Lysis Buffer of red blood cells (RBC) (1X) from BioLegend^®^, mononuclear cells (MNC) were isolated by density gradient using Ficoll-Paque™ Plus from GE Healthcare, and mesenchymal stromal cells (MSC) were derived by culturing MSCs in Dulbecco’s Modified Eagle Medium (1x) from Gibco™ with 10% fetal bovine serum (FBS) from Gibco™ and 1% antibiotic-antimycotic (100X) from Gibco™. Once the cultures reached 80% confluence, MSC were recovered with trypsin-EDTA (0.25%) (Gibco™). PB samples were collected in BD Vacutainer^®^ tubes with EDTA (purple cap) and without anticoagulant (red cap), which allowed whole blood, plasma, leukocytes, and serum to be collected and stored. PB samples were processed in a similar manner as MO samples to obtain whole blood (WB), plasma (P), leukocytes (L), and serum (S). Urine: The samples were labeled and stored without further processing. Prior to conservation, the WBM, WB, MNC, MSC, and L samples were mixed with SFB and 10% dimethyl sulfoxide (DMSO) from Sigma-Aldrich. Some leukocytes were stored in the lysis buffer (LB), whereas others were cryopreserved buffer-free (BF). All samples were stored in a deep freezer at -80°C.

Processing involves four different laboratories, all following rigorously approved protocols that ensure the integrity of the samples, ranging from processing to labeling, conservation, and biosafety. A form was implemented to generate a database in real time to track and document each stage of the Cell Storage Database: It includes the type of sample, stored component, processing date, quantity, status of origin, hospital of origin, name of the storage box, and exact storage coordinates. This meticulous process has resulted in valuable cell collection, providing essential samples from patients with Acute Leukemia for various research projects.

### 2.9 Allocation of specimens to research initiatives

The purpose of this stage is to facilitate laboratories that need to access patient samples and clinical data for their research. To request samples and/or data, it is essential to contact the responsible researcher and sign a confidentiality commitment letter that ensures that both the data and samples will be used exclusively for research purposes.

### 2.10 Collaborative research

The use of digital platforms is crucial in the roadmap, and all data at each stage are entered and updated through digital forms, allowing real-time monitoring and guaranteeing efficient management. All the information generated in each process is concentrated in digital databases that allow the clinical information, test results, reports generated, and other relevant data to be stored and managed in a secure and organized manner, facilitating access to the information by the patients. Specialists involved in the project as well as collaboration and data exchange between different participating hospitals and laboratories, which allows epidemiological studies, trend analysis, and identification of risk factors.

The databases that originated from each procedure were updated and expanded using Google Forms. A specialist periodically monitored these databases to detect and correct any inconsistencies in recorded information. In addition, a backup copy of the information was made every three weeks to guarantee its preservation. Regarding computer security measures, each computer used was protected with passwords, and these computers were placed in a secure room with a biometric access system.

## 3 Results

From March 2022 to August 2023, the Epidemiology Group of the PRONAII Childhood Leukemia identified 346 suspected cases of childhood leukemia. The Childhood Cancer Cytomics Laboratory analyzed 281 cases, accounting for an 81.2% rate of participation (**Table 2**). Consequently, 18.8% of the cases were not evaluated, predominantly due to the inadequacy of the samples provided. Other specific reasons for these losses are detailed in **Table 3**. Sample shipping and processing have an approximate cost of $60 USD, including shipping services, local transportation, and pre-analytical sample processing. The cost of immunophenotyping for diagnosis or MRD assessment ranges from $449 to $1099 USD, depending on each clinical case.

**Table 2:** Rate of participation for leukemia diagnosis confirmation analysis of the study population attended at public hospitals of Oaxaca, Puebla and Tlaxcala during March 2022 to August 2023.

State	Total number of patients with suspected acute leukemia from March 2022 to August 2023	Samples sent to the Childhood Cancer Cytomics Laboratory for leukemia diagnosis confirmation analysis	Participation rate (%)
**Puebla**	218	179	**82.1**
**Oaxaca**	109	83	**76.1**
**Tlaxcala**	19	19	**100.0**
**Global**	**346**	**281**	**81.2**

**Table 3:** Factors contributing to non-assessment of initial bone marrow samples (at diagnosis) in the laboratory.

Reasons	n	%
Insufficient sample	**19**	**29**
Flow Cytometer technical issues	**14**	**22**
Shipment/processing of the samples inactive at weekends and national holiday days	**13**	**20**
Diagnosis samples previously assessed in other Institution (private, other state, etc.)	**6**	**9**
Parents did not consent to participate in the study/Refused treatment initiation	**5**	**7**
Clinicians prefer to send the samples to a private laboratory	**3**	**5**
Samples were not possible to collect due to the critical condition of the patient	**3**	**5**
The laboratory was closed due to intense volcanic emissions of the Popocatepetl	**2**	**3**
**Total**	**65**	**100**

**Figure 2:**
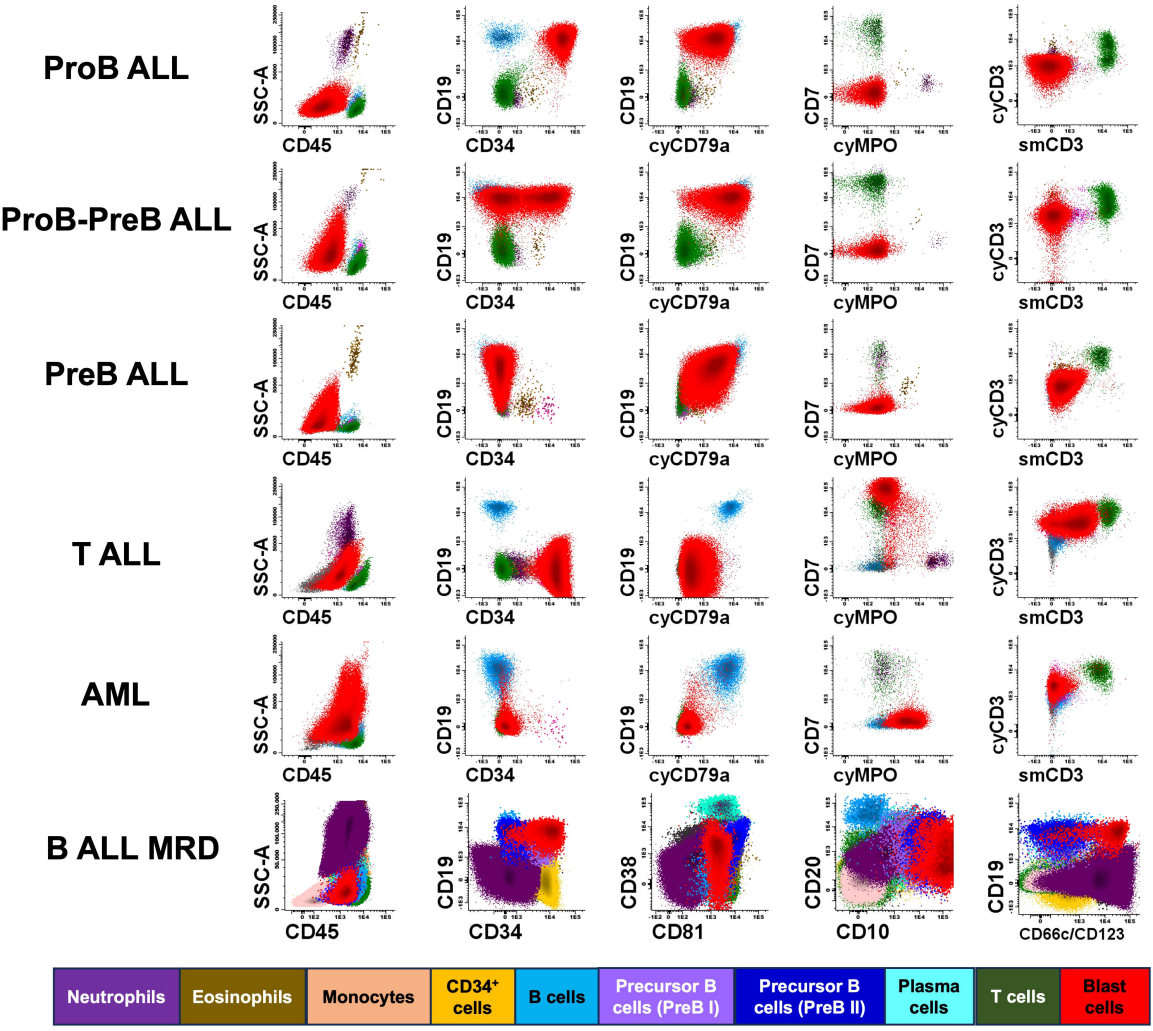
Immunophenotyping of acute leukemia using flow cytometry. This figure presents illustrative cases of various hematopoietic populations, including residual normal and pathological hematopoietic cells detectable in bone marrow samples from pediatric patients with different subtypes of acute leukemia, including ProB ALL, ProB-PreB ALL, PreB ALL, T-cell precursor acute lymphoblastic leukemia (T ALL), acute myeloid leukemia (AML), and detectable B ALL MRD. At diagnosis, dot plots of AL cases showed a blast population comprising more than 20% of nucleated cells, while in the B-ALL MRD test a representative plot detecting only 0.01% of blast is showed. Colors corresponding to each cell population are indicated at the bottom of the figure.

Multiparametric flow cytometry immunophenotyping is crucial for the diagnosis and identification of posttreatment MRD. Antibody panels and immunophenotyping protocols standardized by the EuroFlow™ consortium were used in the Childhood Cancer Cytomics Laboratory. **Figure 2** presents dot plots illustrating cases of acute leukemia, in which the leukemic cells are shown in red. The immunophenotypic profile was used to classify leukemia, ProB ALL, ProB-PreB ALL, PreB ALL, T ALL, and AML. In detectable B-ALL MRD, it is possible to identify the maturation stages of the normal and regenerating B cell lineages as well as the presence of leukemic cells that persist during treatment.

**Figure 3:**
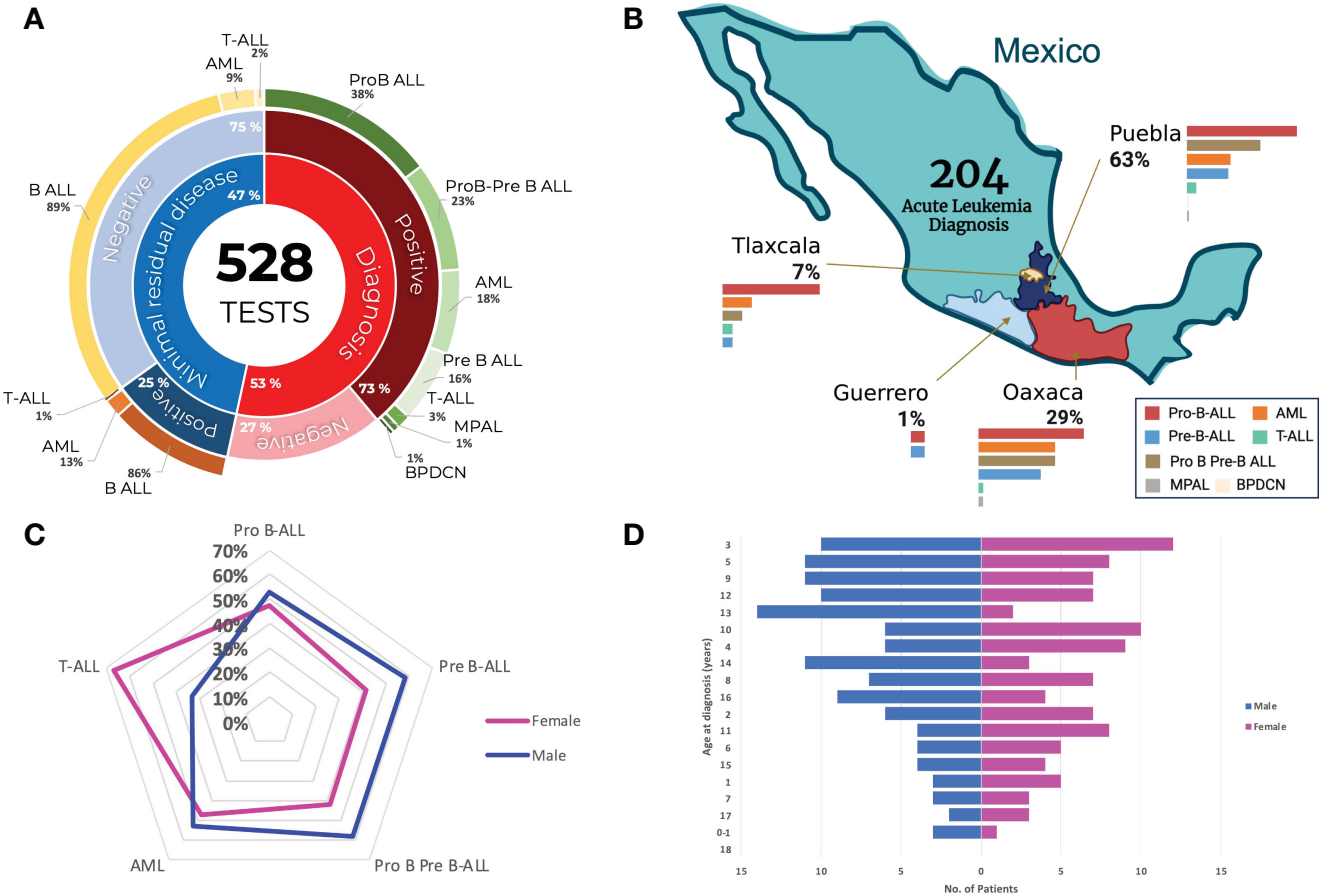
Diagnosis and distribution of acute leukemia in Mexican childhood patients. **(A)** Donut chart detailing all the tests. Inner ring: total and minimal residual disease (MRD) diagnostic tests; second ring: percentage of positive and negative results; third ring: classification of acute leukemia. **(B)** Breakdown of the diagnostic tests performed in the states of Puebla, Oaxaca, and Tlaxcala, showing the frequency according to leukemia classification. **(C)** Radial graph showing the distribution of cases according to sex and acute leukemia subtype. **(D)** Histogram illustrating the number of pediatric acute leukemia cases and their distribution according to age and sex at the time of diagnosis.

Between March 2022 and August 2023, 528 immunophenotype analyses were performed using multiparametric flow cytometry at the Childhood Cancer Cytomics Laboratory. Of the total tests, 53% were diagnostic and 73% were compatible with AL. 47% of the tests were performed to evalute MRD, of which 25% were detectable. The most common types of leukemia diagnosed were ProB ALL (38%), ProB-PreB ALL (23%), and AML (18%) (**Figure 3A**). No marked difference was observed between the type of leukemia and presence or absence of MRD, with the most frequent being Pro B ALL and AML in both cases.

A total of 204 acute leukemia diagnoses were confirmed in Mexican children and adolescents. Of these, 63% belonged to Puebla, 29% to Oaxaca, 7% to Tlaxcala, and 1% to Guerrero. In all states, the most prevalent leukemia was Pro-B-ALL, followed by ProB-PreB-ALL in Puebla. However, in Oaxaca and Tlaxcala, the second most common leukemia subtype was AML (**Figure 3B**). Only two patients were diagnosed in the state of Guerrero. Regarding the distribution of cases by sex, the types of leukemia were more frequent in males; however, cases diagnosed with T-ALL were more frequent in women (**Figure 3C**). The median age at diagnosis was 10 years, the distribution of which is shown in **Figure 3D**.

### 3.1 PRONAII cell repository for childhood leukemia

**Figure 4:**
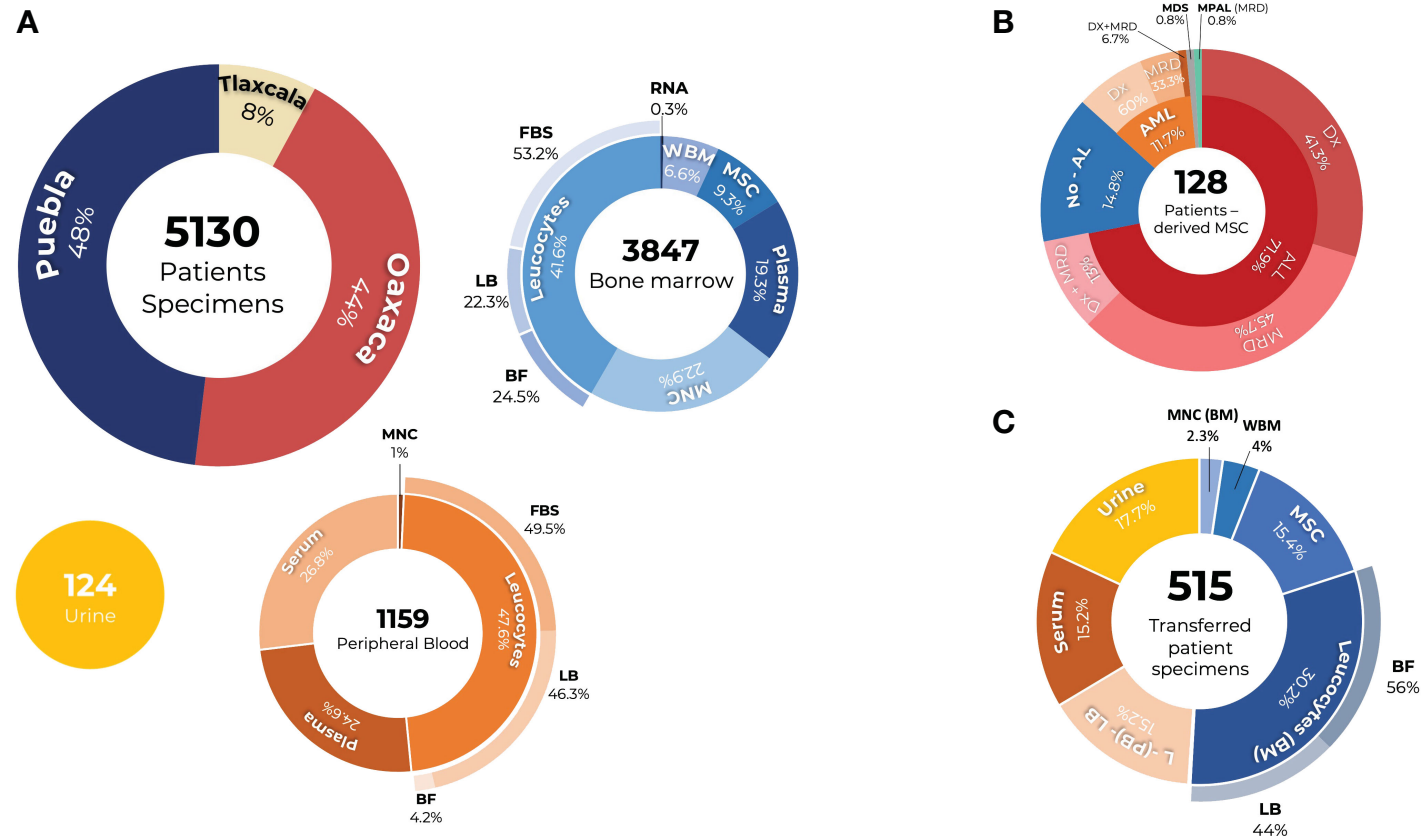
PRONAII Cell Repository of Childhood Leukemia in Mexican Patients: **(A)** A total of 5,130 samples from patients with Oaxaca, Puebla, and Tlaxcala, in which different components of the bone marrow (blue graph) and peripheral blood (orange graph) were preserved, as well as urine samples (yellow graph). Stored components: RNA, ribonucleic acid; plasma, serum; WBM, whole bone marrow; MSC, mesenchymal stromal cells; MNC, mononuclear cells; BF, buffer-free leukocytes; LB, Leukocytes in Lysis Buffer; FBS, leukocytes stored in fetal bovine serum (FBS). **(B)** MSCs were obtained from 128 patients. These include patients with ALL and AML, both at diagnosis and at the minimal residual disease (MDR) stage, as well as those with mixed-phenotype acute leukemia (MPAL) and myelodysplastic syndromes (MDS) at diagnosis. Additionally, MSCs from patients with a negative diagnosis of acute leukemia (non-AL) were stored. **(C)** Different research projects have received 515 specimens from patients.

This collection offers a complete and diverse perspective on the biological profiles of pediatric patients. There was a cell collection of 5,130 specimens, most of which came from the states of Puebla and Oaxaca. Regarding the typology of the samples, 72% were BM samples, 24% were PB samples, and 4% were urine samples. Of the samples obtained from the BM, the majority were leukocytes, MNC, plasma, MSC, and WBM. PB samples included leukocyte, plasma, and serum samples (**Figure 4A**).

From the analysis of the BM samples, we established a unique collection of MSCs derived from 128 patients. The composition of this collection reflects the diversity of leukemic conditions, distributed as follows: 71.9%, ALL cases; 14.8%, acute leukemia (non-AL); 11.7%, AML; 0.8%, MDS; and 0.8%, MPAL. In addition, MSCs obtained from samples were available both at the time of diagnosis and in the MDR evaluation of the same patient, which applies to 13% of patients diagnosed with AL (**Figure 4B**). To date, there are no reports of a bank of MSCs derived from the BM of patients with childhood leukemia in Mexico. Therefore, this PRONAII cell repository represents the first of its kind in the country and is available as a resource to advance research on the leukemic microenvironment.

The PRONAII Childhood Leukemia Cell Repository has contributed significantly to various research projects. These projects have used cell collections for various purposes, including the evaluation of new diagnostic and prognostic tools, searching for possible therapeutic targets, conducting genomic studies, analyzing snoRNA expression, evaluating new pharmacological targets, and studying the tumor microenvironment, such as the creation of predictive profiles and how environmental contaminants affect the hematopoietic microenvironment. Of the total collection, 515 sample components were provided for these studies, including urine, serum, leukocytes, MSC, MNC, and WBM (**Figure 4C**).

## 4 Discussion

Before the implementation of the new diagnostic roadmap, hospitals lacked for a standardized approach for childhood leukemia diagnosis classification using the information derived from the immunophenotype. Particularly, the response to treatment assessment through MRD were carried out in only two hospitals. In this context, the Childhood Cancer Cytomics Laboratory has emerged as a pivotal entity for unifying immunophenotyping results. Moreover, approximately one year before the roadmap’s inception, St. Jude Total XV treatment protocol was adopted across different hospitals, leading to the establishment of individualized care guidelines that incorporated specific supportive care instructions.

One of the main results of the roadmap was to generate and deliver to each hematologist/oncologist a detailed report with the immunophenotype and MRD results of their patients within a period of no more than 72 h from the arrival of the sample to the laboratory. In this regard, it is well recognized that the combination of diagnostic precision and rapid delivery of reports is essential for facilitating opportune and personalized therapeutic decisions (25). According to the results from the epidemiology group, which is also part of this national strategy, one year before the implementation of the roadmap (2021) the average time for treatment initiation after patient arrival to the hospital was 12 days (27). At this moment (2023), treatment initiation has been reduced to six days on average in all participating hospitals. This may particularly be the result of accounting with the information of the immunophenotype in less than 72 hours from the BM collection at diagnosis helping to classify and start treatment in the patients with leukemia.

Biobanks not only serve as repositories of biological samples, but also guarantee their quality and authenticity, which is essential for obtaining reliable results in biomedical research (31). The Biobank of the Autonomous University of Guerrero (UAGro) plays a vital role in providing serum samples from pediatric patients with leukemia. On the other hand, the Leukemic Cell Research Biobank at the Federico Gómez Children’s Hospital of Mexico provides an invaluable resource by storing not only serum but also leukocytes from the BM and PB of pediatric patients with ALL. It is essential that these biobanks continue their work, and that the creation of more of these centers in Mexico and other parts of the world is promoted to facilitate the advancement of research on leukemia and other diseases (32). Access to a wide variety of samples from patients with different subtypes of leukemia and at different stages of the disease is essential to understand the underlying biology and develop more effective treatments (33). The PRONAII Childhood Leukemia Cell Repository stands out in Mexico as it houses a diverse array of biological components from individual pediatric leukemia patients.

In 2013, the Hospital General-Tijuana implemented the WHO Framework for Action, seeking to strengthen its health systems and improve care for ALL. They evaluated the clinical and survival data of children with ALL between 2008 and 2017, distinguishing between the pre- and post-program implementation periods. Thanks to this strategy, a department specializing in leukemia was established, implementing continuous training programs, projects aimed at clinical improvement, and guaranteeing financing for medications, supplies, and personnel through local alliances. After implementation, the 5-year survival rate of children with ALL increased from 59% to 65%. In particular, for children with standard-risk ALL, survival increased from 73% to 100%, and for those at high risk, from 48% to 55%. This transformation resulted in a substantial improvement in the care and survival of patients with ALL in a public hospital in Mexico (34).

Rigorous adherence and management of the childhood leukemia incidence roadmap has allowed for the first time in the region to influence diagnosis confirmation, initial classification, and monitoring of the disease in patients with leukemia by supporting extended immunophenotyping and MRD detection studies, which are currently considered among the most accurate tools for diagnosis, treatment assignment, and response monitoring to chemotherapy. This strategy also impacts in a very relevant way, since by promoting adequate allocation and monitoring of treatment in accordance with national and international recommendations, the damage to patients is limited, such as chemotherapy toxicity and relapses, and reduces the number of deaths and deaths related to disease treatment (23, 35, 36). Additionally, in collaboration with the PRONAII Epidemiology Group, this mechanism is aimed at researching modifiable risk factors associated with disease development. With cell collection, multiple additional studies have been conducted, including genetic, microenvironmental, and immunological studies, contributing to the advancement of comprehensive knowledge in the field of pediatric oncology.

The project encountered several challenges, notably the COVID-19 pandemic, which exerted a significant impact in 2021 and disrupted our implementation roadmap because the priority of that time was the clinical attention of patients with moderate and severe COVID-19, and no research studies that included biological sample collection were allowed by the participating hospital authorities to stop the spread of the virus. Additionally, the initial test phase revealed considerable administrative hurdles, including delays in obtaining ethics committee approval from participating hospitals. Furthermore, to ensure protocol compliance among the roadmap, nearly 100 collaborators were particularly challenging in terms of the timing of sample collection. Therefore, we conducted meetings to emphasize the critical nature of adhering to set deadlines. However, in infrastructure, we were initially equipped with only one flow cytometer. However, we successfully expanded our capabilities using two additional units. The scarcity of personnel for sample processing, exacerbated by the post-pandemic increase in case volume, necessitates the expansion of our team. We also encountered issues with sample quality and quantity; occasionally, we received incorrect samples, such as non-bone marrow specimens. Urgent cases often yielded samples that were inadequate for thorough analysis. Moreover, managing patients who presented with a preliminary leukemia diagnosis without a detailed classification posed a significant barrier to performing accurate comparative analyses.

The forthcoming strategic step involves initiating the formal accreditation process for the Childhood Cancer Cytomics Laboratory in accordance with the ISO 15189 standard, utilizing a Mexican accreditation body, specifically the entidad mexicana de acreditación (ema). This effort is crucial to achieving the goal of ensuring that every patient receives an accurate and timely diagnosis. With certification, the laboratory will not only comply with national and international quality standards but will also reinforce its credibility and reliability in clinical diagnosis. In addition, continuous improvements are planned in diagnostic protocols and processes as well as in cell collection to remain at the forefront of technological innovations and emerging best practices.

This initiative has significantly improved the diagnostic capacity of leukemia in girls, boys, and adolescents in the regions of Oaxaca, Puebla, and Tlaxcala, providing comprehensive, high-quality care with full coverage in the region. Likewise, it has strengthened collaboration between health institutions, researchers, and professionals in the sector, which contributes to reducing the impact of the disease on the community.

## Data availability statement

The raw data supporting the conclusions of this article will be made available by the authors, without undue reservation.

## Ethics statement

The studies involving humans were approved by National Scientific Research and Ethics Committee of the Mexican Institute of Social Security, with the numbers: R-2020-785-177 and R-2020-785-022. The studies were conducted in accordance with the local legislation and institutional requirements. Written informed consent for participation in this study was provided by the participants’ legal guardians/next of kin.

## Author contributions

JN: Conceptualization, Funding acquisition, Methodology, Resources, Visualization, Writing – review & editing. RR: Methodology, Visualization, Writing – review & editing, Data curation, Formal analysis, Investigation, Validation. PG: Data curation, Formal analysis, Investigation, Writing – review & editing, Software. GZ: Investigation, Writing – review & editing. LT: Investigation, Writing – review & editing, Writing – original draft. JA: Investigation, Writing – review & editing, Writing – original draft. JL: Writing – review & editing, Writing – original draft, Data curation, Software. LA: Writing – review & editing, Writing – original draft, Investigation. LL: Investigation, Writing – review & editing. AR: Investigation, Writing – review & editing. DA: Investigation, Writing – review & editing. CT: Investigation, Writing – review & editing. DR: Investigation, Writing – review & editing, Validation. AC: Investigation, Writing – review & editing. JF: Investigation, Writing – review & editing, Software. NL: Writing – review & editing, Resources. AM: Resources, Writing – review & editing. KM: Resources, Writing – review & editing. AR: Resources, Writing – review & editing. JS: Resources, Writing – review & editing. PZ: Resources, Writing – review & editing, Writing – original draft. VT: Resources, Writing – review & editing. ÁM Resources, Writing – review & editing. MG: Resources, Writing – review & editing. RH: Resources, Writing – review & editing. DO: Resources, Writing – review & editing. CC: Resources, Writing – review & editing. EA: Resources, Writing – review & editing. LC: Resources, Writing – review & editing. WH: Resources, Writing – review & editing. BG: Resources, Writing – review & editing. LC: Resources, Writing – review & editing. CE: Resources, Writing – review & editing. GJ: Resources, Writing – review & editing, Investigation. JB: Writing – review & editing, Conceptualization, Methodology. MB: Conceptualization, Methodology, Writing – review & editing, Funding acquisition, Resources. MC: Conceptualization, Writing – review & editing. EÁ: Conceptualization, Writing – review & editing. SP: Writing – review & editing, Funding acquisition, Methodology, Resources. DC: Methodology, Writing – review & editing, Conceptualization, Data curation, Formal analysis, Investigation, Project administration, Supervision, Visualization, Writing – original draft. RP: Conceptualization, Methodology, Project administration, Supervision, Visualization, Writing – original draft, Funding acquisition, Resources.
